# CRISPR/Cas9-mediated Disruption of Fibroblast Growth Factor 5 in Rabbits Results in a Systemic Long Hair Phenotype by Prolonging Anagen

**DOI:** 10.3390/genes11030297

**Published:** 2020-03-11

**Authors:** Yuxin Xu, Hongmei Liu, Huilin Pan, Xinyue Wang, Yuxin Zhang, Bing Yao, Nannan Li, Liangxue Lai, Zhanjun Li

**Affiliations:** 1 Jilin Provincial Key Laboratory of Animal Embryo Engineering, Jilin University, Changchun 130062, China; xuyuxin.hi@163.com (Y.X.);; 2 CAS Key Laboratory of Regenerative Biology, South China Institute for Stem Cell Biology and Regenerative Medicine, Guangzhou Institutes of Biomedicine and Health, Chinese Academy of Sciences, Guangzhou 510530, China

**Keywords:** FGF5, rabbit, hair cycle, long hair

## Abstract

Hair growth and morphology are generally regulated by the hair cycle in mammals. Fibroblast Growth Factor 5 (FGF5), which is a hair cycle regulator, has a role in regulating the hair cycle during the transition from the anagen phase to the catagen phase, and a hereditary long hair phenotype has been widely reported when *FGF5* is mutated in humans and other species. However, there has been no such report in rabbits. Thus, the first exon of rabbit *FGF5* was disrupted by the CRISPR/Cas9 system, and the phenotype of *FGF5^-/-^* rabbits was characterized while using hematoxylin and eosin (H&E) staining, immunohistochemistry, quantitative PCR, scanning electron microscopy, and western blotting. The results showed a significant and systemic long hair phenotype in the *FGF5^-/-^* rabbits, which indicated that *FGF5* is a negative regulator of hair growth. In addition, a decreased diameter of the fiber and a higher area proportion of hair follicle clusters were determined in *FGF5*^-/-^ rabbits as compared with the WT rabbits. Further investigation verified that prolonging the anagen phase in rabbits, with decreased BMP2/4 pathway signaling and increased VERSICAN pathway signaling, caused the systemic long hair phenotype. Taken together, these results indicate a systemic long hair phenotype by prolonging anagen in *FGF5^-/-^* rabbits, which could be widely used for Fur production and an ideal model for studying the mechanism of long hair in the future.

## 1. Introduction

The rabbit is an important livestock species that provides a variety of products, including fiber, meat, and hides. Rabbit hair is one of the preferred natural fibers used in textile industries [1]. In rabbits, hair follicles are structured into groups that usually consist of one central primary hair follicle that produces guard hairs; the primary follicle is surrounded by 2–4 lateral primary hair follicles, which produce awn hairs, and by 20–50 secondary hair follicles, which produce down hairs [2]. In addition, the down hair length is a critical economic trait in wool production, as it is closely associated with wool productivity and yield. The molecular mechanisms regulating rabbit hair growth have remained elusive, although hair fiber length is an important economic trait of rabbits in fur production [1].

Hair is produced from follicles as skin appendages that are unique to mammals, and hair is characterized by periodic regrowth [3,4]. The hair growth cycle can be typically defined as three phases: anagen, the stage during which a new hair is produced; catagen, the stage during which hair elongation ceases; and telogen, the stage during which the follicle is at rest [3]. The putative ‘hair cycle clock’ is thought to be composed of one or several activator/inhibitor pairs that act to adjust and control cycle phase transitions at set thresholds of their activities [5,6]. Studies have demonstrated that mutations or the disruption of *FGF5* are associated with a long hair phenotype in humans [7] and other species, such as mouse [8], cat [9], dog [10], alpaca [11], Syrian hamster [12], and cashmere goat [13,14]. One study also demonstrated the relationship between Hr (hairless) and FGF5 in cetaceans [15]. Most animals with a long hair phenotype have no obvious structural abnormalities, and the follicles remaining in the anagen phase cause the observed increase in hair length [13,16]. During the anagen phase, hair is thought to grow as a result of the proliferation of outer root sheath cells (ORSCs) that are induced by humoral factors that are synthesized and released by dermal papilla cells (DPCs). Further in vitro results have demonstrated that *FGF5* has a role in inhibiting hair growth and inducing catagen by blocking the activation of DPCs [7,17,18]. After the blocking effect, it appears that *FGF5* regulates the anagen to catagen transition by interacting with members of other growth factor families (BMP, TGF-β, EGF, VERSICAN, WNT, NOTCH, and SHH) [18,19,20,21,22]. However, little of this regulation has been validated in vivo.

Mouse models have been widely used to model abnormal hair development and even hair follicle morphology in a three-dimensional (3D) and stochastic way [6,20]. However, mouse models cannot fully recapitulate human phenotypes due to the differences in physiological traits and gene expression between mice and humans [23]. In addition, the comparison of hair follicle morphology in mice and rabbits showed that rabbits have a higher density of hair follicles, a higher follicular orifice percentage in the skin per cm^2^, a higher follicular infundibulum volume, and a larger follicular infundibulum surface [24]. However, to date, no studies have indicated the disruption of *FGF5* in rabbits by gene targeting. Based on this, the disruption of *FGF5* in rabbits will benefit agricultural achievements and generate a more obvious hair phenotype than that in mice.

In this study, for the first time, we generated *FGF5*^-/-^ rabbits via CRISPR/Cas9. Significant long hair fibers with rapid growth and decreased diameters were observed in different regions of fur of *FGF5*^-/-^ rabbits. We also discovered a sex-dominant pattern of growth in *FGF5*^-/-^ and WT rabbits. In addition, the results of this study show that disrupting *FGF5* in rabbits improved the ratio of hair follicle clusters area, but produced no significant difference in the number of hair follicles. Altogether, this study suggests that rabbits with *FGF5* disruption can produce excellent longer hair over their whole bodies, which will provide an ideal model to the Fur industry and study the molecular mechanism of hair cycle in the future.

## 2. Materials and Methods

### 2.1. Ethics Statement

The rabbits that were involved in these experiments were New Zealand white. All of the animal studies were conducted according to experimental practices and standards that the Animal Welfare and Research Ethics Committee approved at Jilin University. New Zealand white rabbits were housed under the 12-hour light-dark cycles in individual cages, and fed twice daily with a basic commercial rabbit diet and water ad libitum.

### 2.2. Construction and in vitro Transcription of Cas9 and sgRNAs

The Cas9 plasmid and constructed sgRNAs were transcribed in vitro, as previously described [25]. Briefly, the Cas9 expression construct (Addgene ID: 48137), which was synthesized and cloned into the vector that was linearized by NotI, was transcribed in vitro while using a mMessage mMachine SP6 TranscriptionKit (Ambion, Austin, the United States) and a RNeasy Mini Kit (Qiagen, Valencia, USA) to purify the mRNA.

Complementary oligonucleotides, 20-nt guide sequences, were annealed at 95 °C for 5 min. and then ramped down to 25 °C to clone into the BbsI-digested pUC57-Simple vector (Addgene ID 51306) under the activity of the T7 promoter. PCR products, which were amplified by T7-F: (50-GAAATTAATACGACTCACTATA-3’) and T7-R: (50-AAAAAAAGCACCGA CTCGGTGCCAC-30), were used for in vitro sgRNA transcription (T7 RNA Synthesis Kit (Ambion)). The sequences were purified while using a TIANquick Midi Purification Kit (DP204) (TIANGEN Biotech, Beijing, China). The transcribed sgRNAs were then purified with a miRNeasy Mini Kit (Qiagen), according to the manufacturer’s instructions. The concentration and quality of RNAs were determined with a NanoDrop 2000 (Thermo, Waltham, MA, USA) and by agarose gel electrophoresis, respectively.

### 2.3. Microinjection and Embryo Transfer

Zygotes were collected from sexually mature New Zealand rabbits, as previously described [26]. The oviducts were flushed with 5 mL of DPBS-BSA, and pronuclear-stage embryos were then collected. Mixtures of gRNAs (40 ng/μL) and Cas9 (200 ng/μL) were microinjected into the cytoplasm of the embryos, and they were then transferred to EBSS medium for short-term culture at 38.5°C, 5% CO_2_, and 100% humidity conditions. Approximately 30–50 injected zygotes were transferred into the oviducts of recipient rabbits.

### 2.4. Genotyping

Genomic DNA from *FGF5* knockout and WT rabbits was isolated using a TIANamp Genomic DNA Kit (TIANGEN, Beijing, China), according to the manufacturer’s instructions. Supplementary Table S2 lists the PCR primers that were used for amplifying mutation detection. PCR products, which were gel purified with a TIAN Gel Midi Purification Kit (TIANGEN, Beijing, China), were cloned into pGM-T vectors (Tiangen, Beijing, China) for sequencing, and at least ten positive plasmid clones were Sanger sequenced.

### 2.5. Histopathology and Immunohistochemistry

Hematoxylin and eosin (H&E) staining and immunohistochemistry were performed, as previously described [26]. The skin tissues from *FGF5* knockout and WT rabbits were fixed with 4% paraformaldehyde for 48 h, embedded in paraffin wax, and then sectioned. The skin sections were stained with hematoxylin and eosin (H&E) and analyzed by microscopy (Nikon ts100, Nikon Corporation, Tokyo, Japan). A primary antibody against *FGF5* (1:200; 18171-AP Wuhan Sanying, China) was used for immunohistochemistry. The slides were imaged with a microscope (ts100; Nikon, Tokyo, Japan) and processed using Photoshop CS5 (Adobe, San Jose, CA, USA).

### 2.6. Scanning Electron Microscopy

Hair samples from the backs of *FGF5^-/-^* and WT rabbits were attached to specimen stubs using double stick conductive tabs, and they were then sputter-coated with gold using a Polaron scanning electron microscope E-1010 (Hitachi, Tokyo, Japan). The samples were imaged using an S-3400N scanning electron microscope (Hitachi, Tokyo, Japan).

### 2.7. RT-qPCR

The protocol for RNA extraction has been described previously [25]. Briefly, the total RNA was isolated from skin that was digested by TRIZOL reagent (TIANGEN, Beijing, China), and then reverse transcription was performed with a FastKing gDNA Dispelling RT SuperMix (TIANGEN, KR118). RT-qPCR was performed while using a BIO-RAD iQ5 Multicolor Real-Time PCR Detection System with Talent qPCR PreMix (SYBR Green) (TIANGEN, FP209). The 2^-ΔΔCT^ formula was used to determine relative gene expression, which was normalized to *GAPDH* mRNA expression. All of the experiments were repeated three times for each gene. All the data are expressed as the mean ± SEM (Standard Error of Mean). Supplementary Table S2 shows the primers used for RT-qPCR.

### 2.8. Western Blot Analysis

Western blot analyses were performed, as described previously [25]. The samples from the back skin of *FGF5^-/-^* and WT rabbits were homogenized and lysed in RIPA buffer supplemented with a protease inhibitor cocktail (Roche, Basel, Switzerland), and they were incubated on ice for 30 min. The antibody against *FGF5* (1:1500; 18171-AP, Wuhan Sanying, China) was used as a primary antibody, while Tublin antibody (1:2000; 10094-1-AP, Wuhan Sanying, China) was used as the loading control.

### 2.9. Prediction of the Modified FGF5 Protein Structure

Two deletions were chosen for analysis to evaluate the effects of targeted deletion. The secondary structures of the truncated proteins were predicted by PHY2 software, and their 3D models constituted by the Phyre server (http://www.sbg.bio.ic.ac.uk/phyre2/html/page.cgi?id=index). The WT forms of the FGF5 and FGF5s proteins are also shown.

### 2.10. Phenotypic Data

The growth curve of down and guard hair was sampled at D40. The hair samples were collected from eight different body sites and from different sexes at D70. The length of down and guard hair was measured using Image Pro Plus 6.0 (Media Cybernetics, Rockville, MD, USA) to analyze a photo of hair taken against a black background with a ruler. Pairwise data were analyzed with Student’s t-test while using GraphPad Prism 8.0 (GraphPad Software Inc., San Diego, CA, USA).

### 2.11. Statistical Analyses

The data were analyzed using GraphPad Prism 8.0 (T test) software (GraphPad Software Inc.). A *p*-value < 0.05 was considered to be statistically significant.

### 2.12. Off-Target Assay

Potential off-target sites (POTS) of the sgRNAs were predicted using the CRISPR online design tool (http://tools.genomeengineering.org). The top five POTS were selected. Primers (Supplementary Table S3) were used to test whether the sgRNAs had off target mutations, and T7E1 assay and Sanger sequencing of cloned products that were both placed into pGM-T vectors analyzed the products (Tiangen, Beijing, China).

## 3. Results

### 3.1. Generation of FGF5^-/-^ Rabbits by the CRISPR/Cas9 System

**Figure 1:**
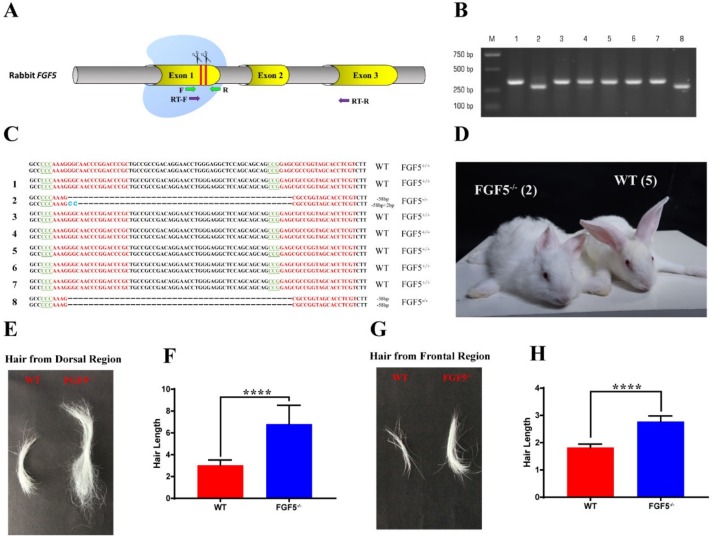
Generation of FGF5^-/-^ rabbits via the CRISPR/Cas9 system. (**A**) Schematic diagram of two sgRNA target sites located in rabbit *FGF5* exon 1. *FGF5* exons are indicated by yellow cylinders. The target sites of sgRNAs are highlighted by red lines and scissors. Green arrows indicate the PCR primers, and purple arrows indicate the RT-qPCR primers. (**B**) Deletion analysis of eight rabbits by PCR using gDNA; M, marker. (**C**) Genotyping by Sanger sequence. PAM is highlighted in green, and target sequences are shown in red; deletions (−); the rabbit number is on the left; WT, wild-type. (**D**) Photographs of F0 *FGF5^-/-^* rabbits and WT rabbits generated by the CRISPR/Cas9 system; the rabbits weighed 0.85 kg and 0.83 kg, respectively. (**E**) and (**G**) Photographs of dorsal hair and frontal hair collected from WT rabbits and *FGF5^-/-^* rabbits, respectively. (**F**) and (**H**) Measurement of hair length from (**E**) and (**G**). The data were analyzed with Student’s t tests using GraphPad Prism software. **** *p <* 0.0001.

The *FGF5* gene was found to be highly conserved (91% identity) between rabbits and humans, as well as at the amino acid sequence level of *FGF5* (Figure S1). Thus, two sgRNAs targeting the first exon of *FGF5* were designed to mimic mutations in human [7], and Figure 1A shows the target sites. 137 injected embryos were transferred to three pseudo pregnant recipient rabbits to generate the *FGF5* deletions in rabbits. One of these recipient mothers was pregnant to term and gave birth to seven live pups and one dead pup (No. 8) (Table S1). Genomic DNA samples from F0-generation pups were extracted and tested for *FGF5* mutations. The T-cloning and PCR-sequencing results revealed that the monoallelic deletion of *FGF5* was present in pups No. 2 (−58 bp, −58 +2 bp) and No. 8 (−58 bp, −58 bp) (Figure 1B,C).

Next, the disruption of *FGF5* could cause a significant long hair phenotype in rabbits, as shown in Figure 1D. We also collected hair from the dorsal and frontal regions, as shown in Figure 1D. Hair length was significantly increased in *FGF5^-/-^* rabbits when compared to hair length in WT rabbits (Figure 1E,G). These results were also confirmed by the measurement of hair length by Imagine Pro, which indicated that the modification of *FGF5* is the main cause of the significant long hair phenotype in the rabbits.

We isolated the *FGF5^-/-^* rabbit genome and selected four potential off-target sites (POTs) for each sgRNA to test whether off-target effects could occur in these rabbits. Table S3 lists the primers and mismatch sites. The POTs were amplified by PCR and Sanger sequenced (Figure S2). The results revealed that none of the POTs were mutated, indicating that the sgRNAs used in this study are locus-specific.

### 3.2. Heritability of the Long Hair Phenotype in FGF5^-/-^ Rabbits

**Figure 2:**
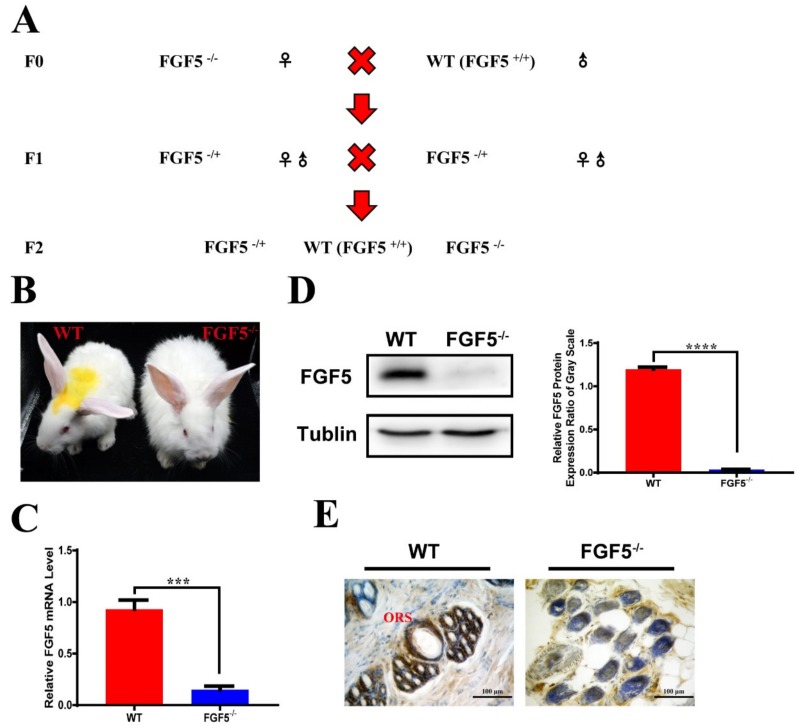
Heritability and analysis of F2 FGF5^-/-^ rabbits. (**A**) Schematic diagram of the generation of F2 *FGF5^-/-^* rabbits. (**B**) Photograph of F2 WT and *FGF5^-/-^* rabbits. (**C**) Expression of *FGF5* was determined by RT-qPCR. (**D**) FGF5 protein levels were determined by western blot (left) and densitometry analysis of the FGF5 protein (right). All of the data are expressed as the mean ± SEM (Standard Error of Mean). *** *p <* 0.001, **** *p <* 0.0001. (**E**) Immunohistochemical staining showed the complete loss of FGF5 protein in the outer root shelter of the hair follicle in *FGF5^-/-^* rabbits.

A female F0 (*FGF5*^-/-^) was mated with a WT rabbit to study the heritability of the long hair phenotype caused by disruption of *FGF5*. The F1 rabbits were all heterozygous, with a deletion of *FGF5*, though the typical symptom of long hair was not observed. A sexually mature F1 was mated within the F1 group to study the heritability of the long hair phenotype caused by disruption of *FGF5* (Figure 2A). Sanger sequence analysis (Figure S3), which demonstrated that deletions of *FGF5* were heritable in rabbits, confirmed the genotypes of F2 generation of *FGF5* knockout rabbits. As expected, *FGF5*^-/-^ F2 rabbits had significantly long hair as compared with WT rabbits (Figure 2B). The *FGF5* mRNA level was significantly reduced in *FGF5*^-/-^ rabbits when compared with WT rabbits, as shown in Figure 2C. Further confirmation of the disruption of *FGF5* was evaluated at the protein level using western blotting (Figure 2D). These results suggested that the nonsense-mediated decay (NMD), the RNA degradation that is used to prevent the accumulation of truncated and potentially harmful proteins, modulated the expression of *FGF5* in *FGF5*^-/-^ rabbit, which is consistent with previous studies [27,28].

A previous study has shown that FGF5 protein is expressed in the outer root shelter [29,30]. As expected, the IHC (immunohistochemistry) results showed that FGF5 protein surrounding the entire follicle was restricted to ORS cells of hair follicles in WT skin, but not in *FGF5*^-/-^ skin (Figure 2E).

### 3.3. The Systemic Effect of Disruption of FGF5 in Rabbit Fur

**Figure 3:**
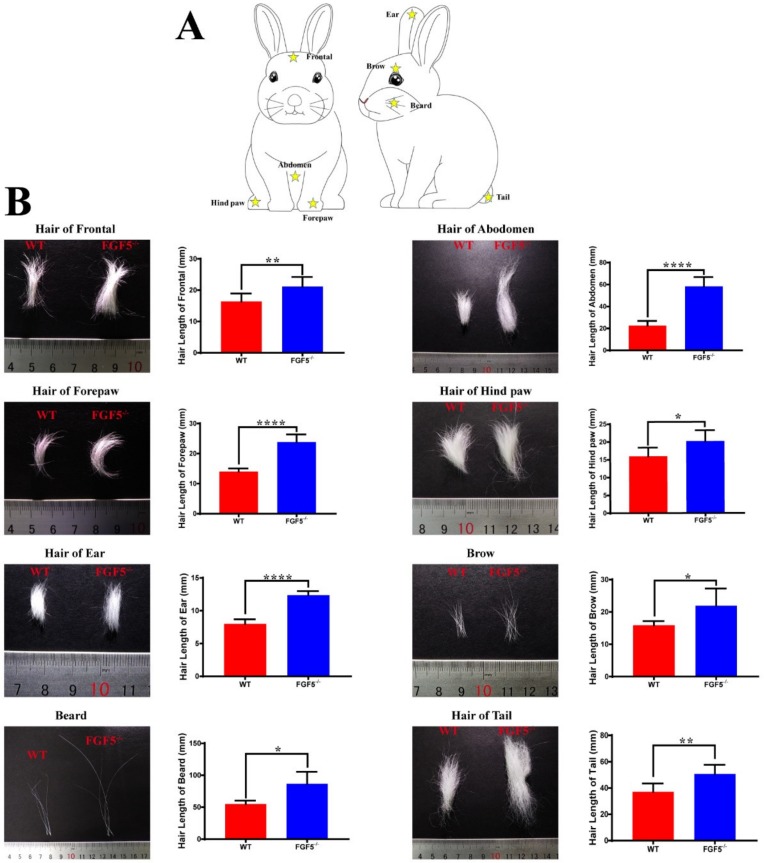
Systematic effect of long hair phenotype. (**A**) Eight parts (frontal, forepaw, ear, beard region, abdomen, hind paw, brow, and tail) of the rabbit were selected for hair collection. Yellow stars indicate the sites of collection. (**B**) Photographs of the hair collected from the eight parts and the hair length between *FGF5^-/-^* rabbits and WT rabbits. All of the data are expressed as the mean ± SEM. * *p <* 0.05, ** *p <* 0.01, **** *p <* 0.0001.

A previous study demonstrated that a mutation in *FGF5* could prolong the growth phase (anagen) of hair [30]. Moreover, angora mice are characterized by possessing a long anagen phase and an increased ratio of anagen:telogen. Therefore, the most obvious effect of a mutation in *FGF5* could be observed on the body sites that usually have short hair with a high proportion of hair follicles in the telogen phase. Thus, hair was collected from eight different body sites of WT and *FGF5^-/-^* rabbits (Figure 3A), and fiber length was recorded and measured, to study whether the disruption of *FGF5* could cause a long hair phenotype in different regions. The results demonstrated that the disruption of *FGF5* resulting in long hair systemically affects all fur (Figure 3B). Interestingly, the hair length in the belly, forepaw, and ear was tremendously increased in *FGF5*^-/-^ rabbits when compared with the WT rabbits. We speculated the lower proportion of anagen:telogen of hair from the belly, forepaw, and ear than hair from other parts of body that were more sensitive to the disruption of *FGF5*. However, more investigation should be carried out in future studies. Altogether, the results suggested that the disruption of *FGF5* protein could cause a systemic long hair phenotype in rabbits.

### 3.4. Sex-Dominant Growth Pattern During Hair Development in FGF5^-/-^ Rabbits

**Figure 4:**
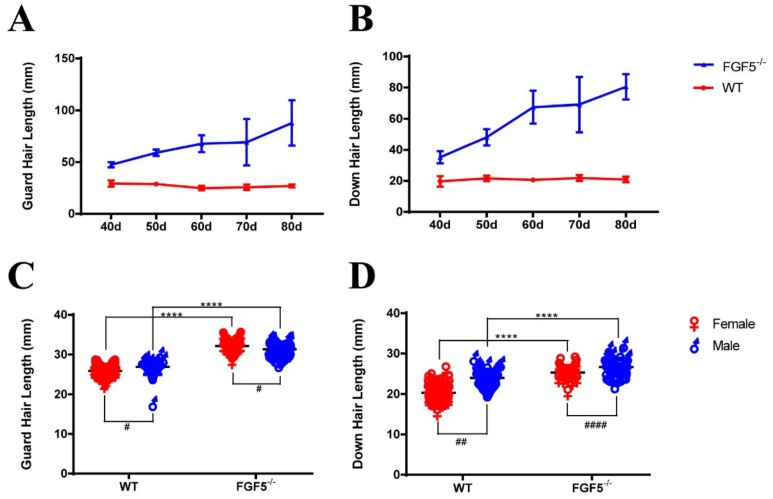
Evaluation of hair length of F2 FGF5^-/-^ and WT rabbits. (**A**) and (**B**) The length of guard hair and down hair compared between FGF5^-/-^ rabbits and WT rabbits. (**C**) The length of guard hair in female and male FGF5^-/-^ and WT rabbits. (**D**) The length of down hair in female and male FGF5^-/-^ and WT rabbits. The length difference of FGF5^-/-^ and WT rabbits in the same sex was presented as * and the length difference of FGF5^-/-^ and WT rabbits in the distinct sex was presented as # All the data are expressed as the mean ± SEM. * *p < 0.05*, ** *p < 0.01*, *** *p < 0.001*, **** *p < 0.0001*.

As *FGF5* is the master regulator controlling hair length, we measured the length of both guard hair and down hair of rabbits, starting from day 40 (D40) after birth, and every 10 days throughout the trial. The length of both guard and down hair had a sharp increase in *FGF5*^-/-^ rabbits, when compared with those of the WT controls, as shown in Figure 4A,B.

Previously, one study has shown that the mutation of *FGF5* in Syrian hamster caused a long hair phenotype with male dominant expression [12]. Thus, the guard and down hair of the *FGF5*^-/-^ and WT rabbits were compared between male and female (Figure 4C,D). These results confirmed that WT and *FGF5*^-/-^ rabbits both had a sex-dominant growth pattern, but female rabbits are more sensitive to FGF5-dependent hypertrichosis in *FGF5*^-/-^ rabbits.

### 3.5. Morphological Characterization of Hair and Skin in FGF5^-/-^ Rabbits

**Figure 5:**
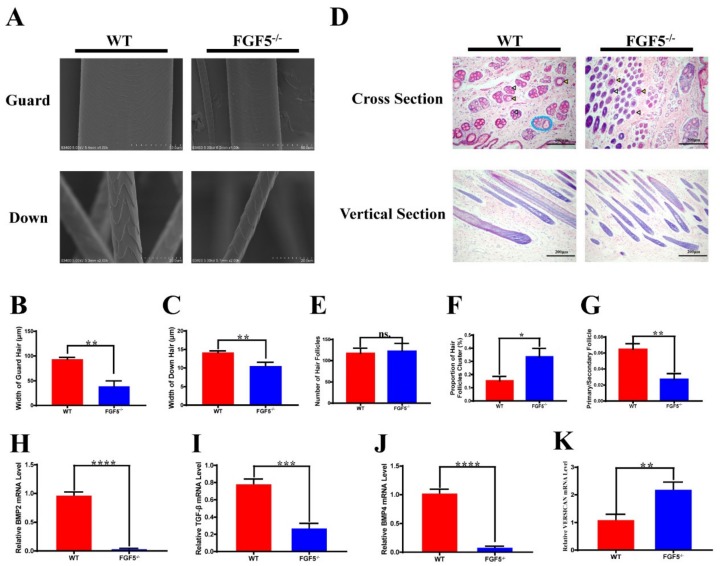
Fiber and pathway differences between FGF5^-/-^ and WT rabbits. (**A**) Scanning electron microscopy (SEM) of guard hair and down hair from *FGF5^-/-^* and WT rabbits. (**B**,**C**) Width of guard and down hair in *FGF5^-/-^* and WT rabbits, respectively. (**D**) Hematoxylin and eosin (H&E) staining of skin from *FGF5^-/-^* and WT rabbits. The yellow triangle indicates the primary hair follicle (PHF), and the white triangle indicates the secondary hair follicle (SHF). The blue oval indicates a classical hair follicle cluster. (**E**–**G**) Analysis of number of hairs, proportion of hair follicle clusters, and primary/secondary follicles, as determined from cross section H&E staining of *FGF5^-/-^* and WT rabbits, respectively. (**H**–**K**) The mRNA levels of hair cycle-related genes: BMP2, BMP4, VERSICAN, and TGF-β.

The key traits contributing to the economic value of rabbit hair include fiber diameter, density, length, and strength. Some modification of hair-related genes can generate fragile hair fibers [25]. A SEM (scanning electron microscope) was used to assess the hair of the WT and *FGF5*^-/-^ rabbits to examine the role of *FGF5* in hair structure and shaft. The SEM result demonstrated that there is no significant difference of hair structure and shaft, while a decreased diameter of the guard and down hair shaft (Figure 5B,C) in *FGF5*^-/-^ rabbits, when compared with those WT controls, as shown in Figure 5A. Further, there was no squama exuviation or occasional longitudinal fissures that were found in the guard and down hair of *FGF5*^-/-^ rabbits. Hematoxylin and eosin (H&E) staining (Figure 5D) was performed to measure the number of hair follicles (Figure 5E), the proportion of hair follicle cluster area (Figure 5F), and the ratio of primary/secondary follicles (Figure 5G). The results demonstrated that the hair follicles of WT rabbits appeared to be contracted, and the area of primary and secondary hair follicles were obviously decreased in comparison to those of *FGF5*^-/-^ rabbits. Moreover, increased secondary follicles, while no obvious difference in the total number of hair follicles, were detected in *FGF5^-/-^* rabbits. Altogether, the *FGF5*^-/-^ rabbit model was equipped with long fibers, fine fibers, and bushy hair follicles, which indicated that *FGF5*^-/-^ rabbits are a new model for high-quality rabbit hair production.

### 3.6. Structural Alterations of FGF5 and FGF5s Protein in FGF5^-/-^ and WT Rabbits

The *FGF5* gene can produce a full-length protein (FGF5) and a short form (FGF5s) through mRNA alternative splicing. *FGF5s* has been noted to suppress *FGF5* activity by competitively binding to the FGF receptor 1, FGFR1 [31]. We performed a prediction of FGF5 and FGF5s protein secondary structures and constructed 3D models for these two major genotypes to precisely assess the putative functional impact of the *FGF5* modification (Figure S4B–E). The predicted protein structure showed that the FGF5 and FGF5s proteins were truncated due to premature termination and that the proteins lost the critical b-strand domain (Figure S4A), which is necessary for *FGF5* signal transduction [32]. This finding suggests that mutations that are induced by CRISPR/Cas9 in this study led to the disruption of *FGF5* and *FGF5s* function.

### 3.7. The Long Hair Genotype in FGF5^-/-^ Rabbits Was Caused by a Classical Pathway

Previous studies have shown that the BMP signaling pathway plays a role in maintaining telogen [19,33]. Similarly, the suppression of TGF-β delays catagen in hair follicles [22]. Finally, VERSICAN is a marker of anagen in the dermal papilla [21]. Thus, we detected the mRNA expression levels of hair growth-related molecules while using quantitative real-time PCR. The mRNA expression levels of *BMP2/4* and *TGF-β* were significantly decreased in *FGF5^-/-^* rabbits, whereas the *VERSICAN* mRNA expression level was increased (Figure 5H–K). These results demonstrate that prolonged anagen caused increased the proliferation of hair follicle cells and a decrease in signals suppressing proliferation. Moreover, *FGF5* has a negative effect on hair follicle growth.

## 4. Discussion

The hair follicle is a special mammalian organ that has continuous cycles, namely, anagen, catagen, and telogen, throughout an organism’s whole life [3]. Studies have begun to elucidate how the niche, the cellular microenvironment, influences hair follicle growth and cycling in fascinating new ways [34]. Some of these studies echo a hypothesis that was reported approximately 50 years ago and championed by William Bullough and colleagues. They described an antimitotic substance (“chalones”) in skin that stimulated epidermal mitosis when it was removed [35]. The inhibitory signal that synchronizes follicle lengthening and shortening was discovered in recent years [34]. The hair cycle will remain in the very phase unless the inhibitory signal relieves. Recently, increasing evidence has shed light on this theory and shows the potential for new directions in studying skin disorders; however, the cross-talk among niches around hair follicles highlights the rich complex research that is yet to be performed.

**Figure 6:**
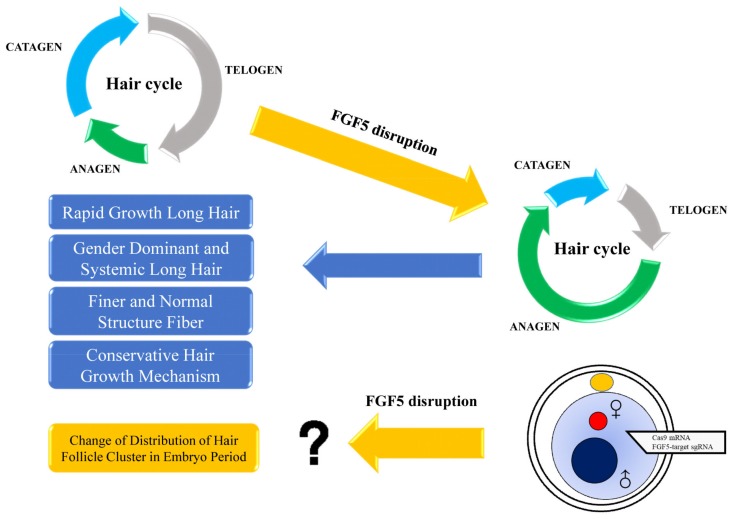
A synopsis of this study. Disruption of *FGF5* could prolong the anagen (growth phase) of the hair cycle, resulting in rapid growth of long hair. In addition, sex-dominant and systemic long hair were determined in *FGF5^-/-^* rabbit. The fiber of the long hair possessed a normal structure, but was finer than WT fibers after the disruption of *FGF5*. These results demonstrated that rabbits could be a useful animal model for high-quality hair industry and for hair cycle study. Interestingly, we assumed that disruption of *FGF5* could change the distribution of hair follicle clusters during the embryonic period. However, more studies should be conducted to investigate this.

*FGF5* is a secreted inhibitory signal, and its function is to inhibit hair elongation [30]. *FGF5* was first studied in long hair mice [8]. The long hair phenotype, which is caused by mutations in *FGF5*, was found in many kinds of species, such as cat [9], dog [10], donkey [36], goat [32], Syrian hamster [12], and mammoth [4]. In this study, the results demonstrated that disruption of *FGF5* contributed to longer anagen, which resulted in rapid growth long hair, gender dominant and systemic long hair as well as finer and normal structure fiber. In addition, the difference of classical pathway in the *FGF5^-/-^* rabbits demonstrated the conservation among different species. However, the function of *FGF5* in distribution of hair follicle cluster in embryo period need to be studied in the future (Figure 6). 

Studies of *FGF5* have shown that the location of *FGF5* is in the outer root sheath cells [30], which is consistent with our immunohistochemical staining results. However, the purple staining in the surrounding cells of hair follicles (Figure 2E) and the significantly decreased while not completely lost band in the Western Blot (Figure 2D) were also determined in the *FGF5^-/-^* rabbits. We assumed that the non-specific staining is due to the fact that there is no commercial high quality monoclonal antibody for rabbit, which is a common issue for rabbit’s protein detection by Western Blot or immune staining. Especially, the high similarity between *FGF5* and *FGF6* has been reported and *FGF6* is also expressed in skin and weighed 22 kDa (*FGF5* weighed 29 kDa) [37]. Thus, the high quality antibody for *FGF5* protein of rabbit is necessary for the identification and investigation of genotype to the phenotype of *FGF5^-/-^* rabbit in the further study.

The secretion of *FGF5* interacts with the receptor FGFR1, which is expressed in dermal papilla cells (DPCs); this signal is known to stimulate outer root sheath cell (ORSC) proliferation [17]. Other molecular factors were also found to play a role in the hair cycle. BMP4 is expressed in dermal papilla and it is involved in the hair growth cycle during the late anagen and telogen phases in mice [19,33]. VERSICAN, which is a chondroitin sulfate proteoglycan with a large molecular mass, is implicated in the maintenance of hair growth in human [21]. The expression of VERSICAN reaches its maximum during anagen and then declines at the end of anagen. TGF-β1 induces the catagen phase of the hair cycle and it can induce morphological changes and apoptosis in cultured human follicles [22]. In *FGF5*^-/-^ rabbits, we found increased *VERSICAN* levels and decreased *BMP2/4* and *TGF-β* levels. The results from the *FGF5*^-/-^ rabbits indicated that rabbits might share a similar mechanism with humans and rodents. This conversed hair development strategy may be very useful for studying abnormal hair growth, such as alopecia and polytrichosis. In the future, rabbits could be an excellent model for hair study due to their size and conversed hair cycle mechanism.

In humans, different body sites, such as the upper, eyelashes, and scalp, have variable percentages of hair follicles in anagen, from low to high, respectively [38,39,40]. Mutations in *FGF5* could cause localized hypertrichosis in human legs, arms, and eyelashes, because these sites have a low anagen:telogen ratio [7]. In our *FGF5^-/-^* rabbit, a systemic long hair phenotype was determined, but the different body sites had different reactions to disruption of *FGF5*. Multi-scale modeling has shown that mouse skin behaves as a heterogeneous regenerative field, in which different regions of the body (fast cycling and slow cycling) have distinct cycling dynamics. Some areas, such as the ear, act as a hyper refractory domain with HFs in the extended rest phase [6]. Therefore, we hypothesize that the belly, ear, and forepaw in rabbits have a low anagen:telogen ratio and they are slow-cycling regions. These results demonstrated that rabbit hair regulation could be more complex than mouse and human hair regulation and may be a model for human hair growth studies. These results also indicate that the systemic long hair phenotype could be a good characteristic of hair yield.

A previous study [8] showed that the mutations of *FGF5* could induce occasional longitudinal hair fissures with broken or curled tips, while other reports suggested that the structure of secondary hair follicles (SHF) was not affected by the disruption of *FGF5* [13]. The SEM results demonstrated that the surface structure of the hair fiber showed no obvious damage, while the width of both the guard and down hair of the *FGF5^-/-^* rabbit decreased. The cross section of skin from *FGF5^-/-^* rabbits showed no obvious change in the number of hair follicles, but there was a change in the size of the hair follicle cluster area and the ratio of primary/secondary hair follicles. The higher ratio of hair follicle cluster that was observed in H&E staining could explain the bushy feeling when we touched the *FGF5^-/-^* rabbits. The decreased primary/secondary follicle ratio demonstrated an increase in the number of secondary hair follicles, which can generate more down hair and would be useful in producing textiles.

The BMP family and SHH balance the fate decision and generation of hair follicles during the embryonic period. In addition, previous work demonstrated that *FGF5* has no obvious effect on hair follicle development in mouse, but have an effect on early chick development [41,42]. Based on the change in the hair follicle cluster, we assumed that the distribution of hair follicle clusters could be regulated by *FGF5* during the development of the embryo, although additional work should be carried out. In conclusion, our results demonstrated that *FGF5^-/-^* induced a long hair phenotype and had effects on the distribution of hair follicles among species.

The disruption of *FGF5* can optimize features of hair fibers by producing long fibers that are thin and have a bushy distribution. These features are very important and useful in hair production. The spontaneous long hair that was caused by mutations of *FGF5* in Syrian hamsters was affected by testosterone administration [12]. Male dominant effect was determined and weakened after the disruption of *FGF5*. Moreover, the prolonged anagen phase could be sustained for a long time.

In this study, for the first time, we generated *FGF5^-/-^* rabbits via CRISPR/Cas9 methodology. This result suggests that rabbits with *FGF5* disruption can produce excellent long hair over their whole bodies, which can be very useful in agricultural areas and studies regarding the hair follicle cycle.

## 5. Conclusions

*FGF5^-/-^* rabbits could be a novel model to promote Fur production and ideal model to investigate mechanism of long hair.

## Supplementary Materials

The following are available online at https://www.mdpi.com/2073-4425/11/3/297/s1, Figure S1. Alignment of amino acid sequences of FGF5 between rabbit and human. Figure S2 Off-target analysis of sgRNAs and sequence diagram of POTs in KO rabbits. Figure S3. Gene editing events of F2 *FGF5*^-/-^ rabbits. Figure S4. Prediction of the structural alteration among predominant FGF5-targeted and WT proteins. Table S1. Generation of genetically targeted rabbits using CRISPR/Cas9. Table S2: Primers used in PCR. Table S3. 8 potential off-target sites examined by PCR and primers used for list.
