# Supplementary material for: CRISPR/Cas9-mediated Disruption of Fibroblast Growth Factor 5 in Rabbits Results in a Systemic Long Hair Phenotype by Prolonging Anagen
Source: Genes (Basel). 2020 Mar 11;11(3):297. doi: 10.3390/genes11030297 (PMC7140871; doi:10.3390/genes11030297)
Supplement: Supplementary file 1 [file genes-11-00297-s001.pdf]

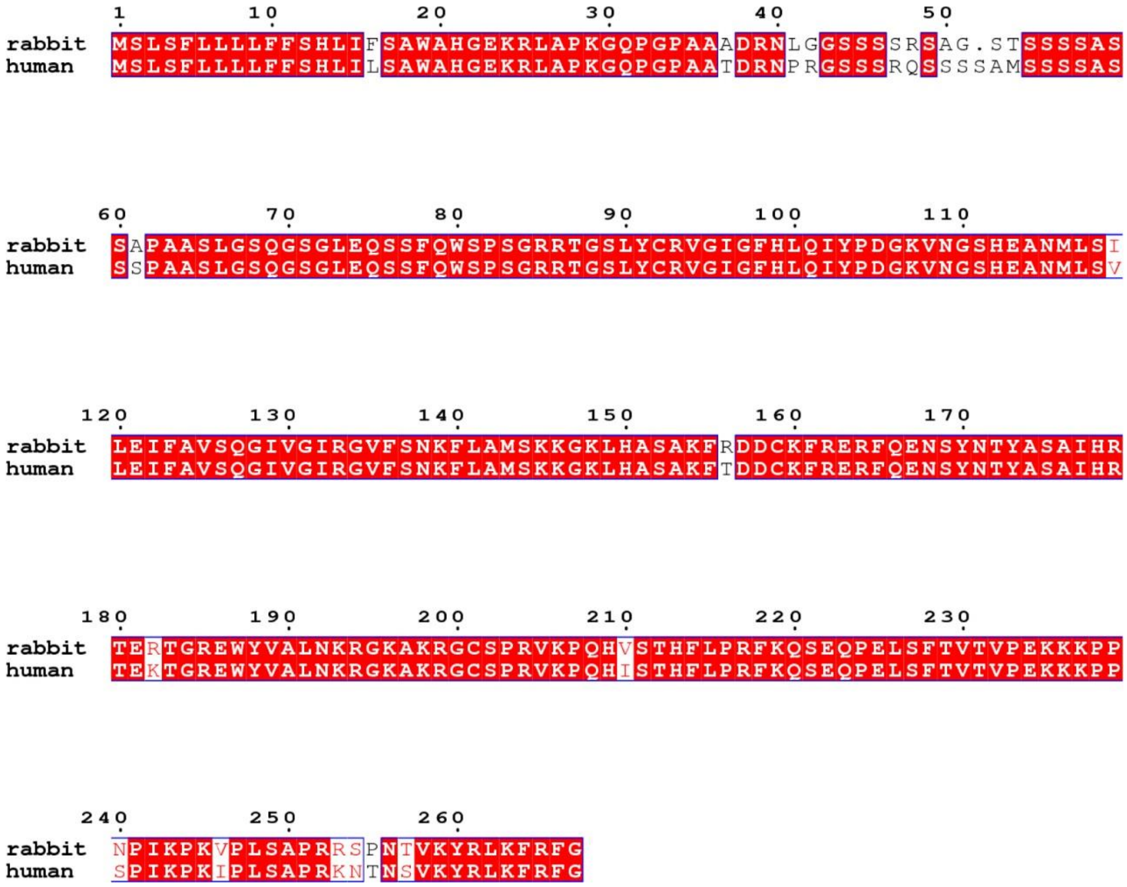

Figure S1. Alignment of amino acid sequences of FGF5 between rabbit and human. Identical amino acids are highlighted in red.

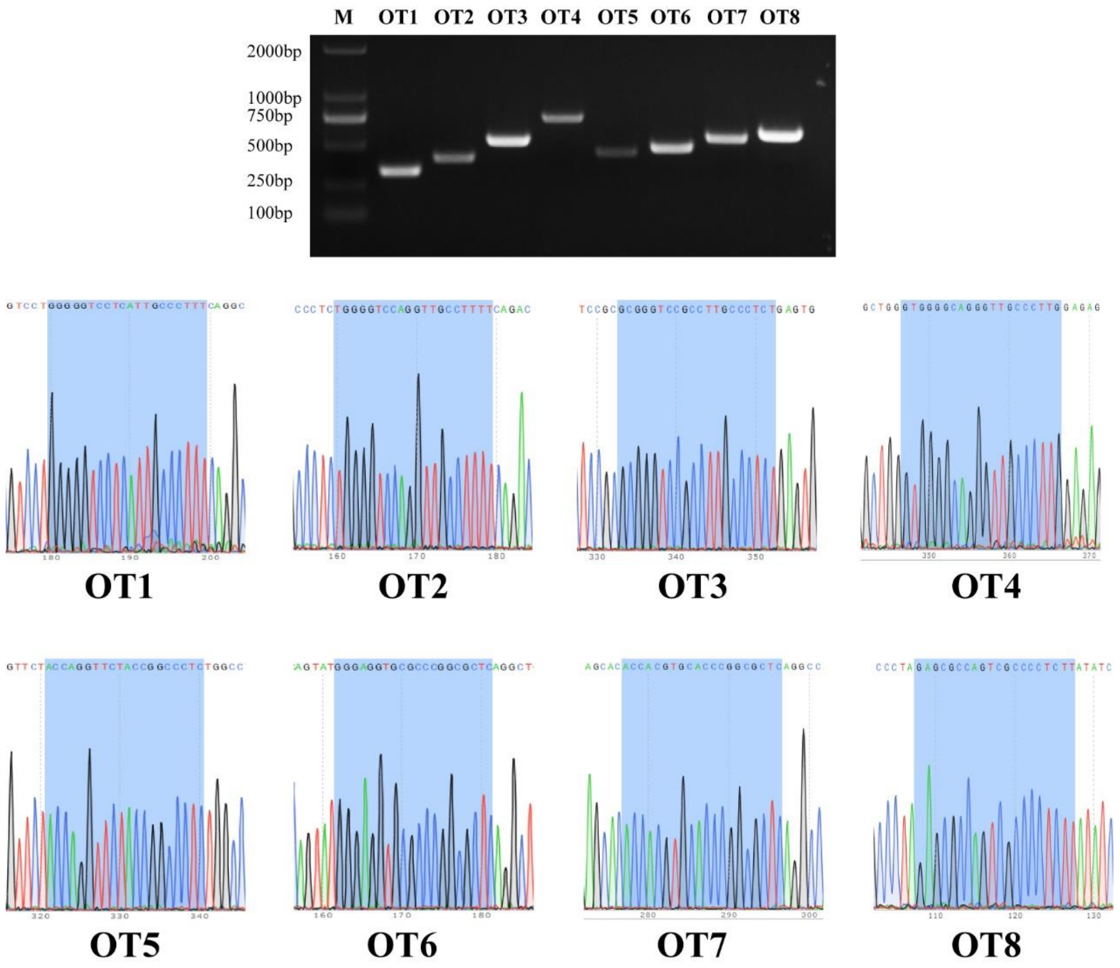

Figure S2 Off-target analysis of sgRNAs and sequence diagram of POTs in KO rabbits.

PCR products of candidate off-target sites for two sgRNAs are analyzed and no fragment are found in gel electrophoresis. Sequence diagram of 10 potential off-target sites is shown and no double curve are found. Blue area represents sequencing of the POTs.

Label	Gene Sequence	Indel Type	Fragment Change		Allelic Modification
#1	GCCCCCAAAGGGCAACCCGGACCCGCTG···32bp···AGCCGGAGCGCCGGTAGCACCTCGTCTT<br>GCCCCCAAAGGGCAACCCGGACCCGCTG···32bp···AGCCGGAGCGCCGGTAGCACCTCGTCTT	Wild Type<br>Wild Type	3N+0; N=0<br>3N+0; N=0	In Frame<br>In Frame	$FGF5^{+}/FGF5^{+}$
#2	GCCCCCAAAG··························CGCCGGTAGCACCTCGTCTT<br>GCCCCCAAAGCC·······················CGCCGGTAGCACCTCGTCTT	-58bp<br>-58bp+2bp	3N+1; N=19<br>3N+2; N=18	Premature Termination<br>Premature Termination	$FGF5^{-}/FGF5^{-}$
#3	GCCCCCAAAGGGCAACCCGGACCCGCTG···32bp···AGCCGGAGCGCCGGTAGCACCTCGTCTT<br>GCCCCCAAAGCC·······················CGCCGGTAGCACCTCGTCTT	Wild Type<br>-58bp+2bp	3N+0; N=0<br>3N+2; N=18	In Frame<br>Premature Termination	$FGF5^{+}/FGF5^{-}$
#4	GCCCCCAAAG··························CGCCGGTAGCACCTCGTCTT<br>GCCCCCAAAG··························CGCCGGTAGCACCTCGTCTT	-58bp<br>-58bp	3N+1; N=19<br>3N+1; N=19	Premature Termination<br>Premature Termination	$FGF5^{-}/FGF5^{-}$
#5	GCCCCCAAAGCC·······················CGCCGGTAGCACCTCGTCTT<br>GCCCCCAAAGCC·······················CGCCGGTAGCACCTCGTCTT	-58bp+2bp<br>-58bp+2bp	3N+2; N=18<br>3N+2; N=18	Premature Termination<br>Premature Termination	$FGF5^{-}/FGF5^{-}$
#6	GCCCCCAAAGGGCAACCCGGACCCGCTG···32bp···AGCCGGAGCGCCGGTAGCACCTCGTCTT<br>GCCCCCAAAG··························CGCCGGTAGCACCTCGTCTT	Wild Type<br>-58bp	3N+0; N=0<br>3N+1; N=19	In Frame<br>Premature Termination	$FGF5^{+}/FGF5^{-}$

Figure S3 Gene editing events of F2 FGF5$^{-/-}$ rabbits. Six genotypes are shown in numbers #1-#6; sequence: the PAM sequence is indicated in green; the target sequence is indicated in red; the insertion sequence is indicated in blue. All modifications were determined by alignment with the WT sequence. Fragment change shows the predicted translation events (premature termination or in frame) resulting from targeting based on exact mutations. Allelic modifications indicate characterization of targeting by biallelic modification or monoallelic modification; +/+, WT; -/-, biallelic modification; +/-, monoallelic modification.

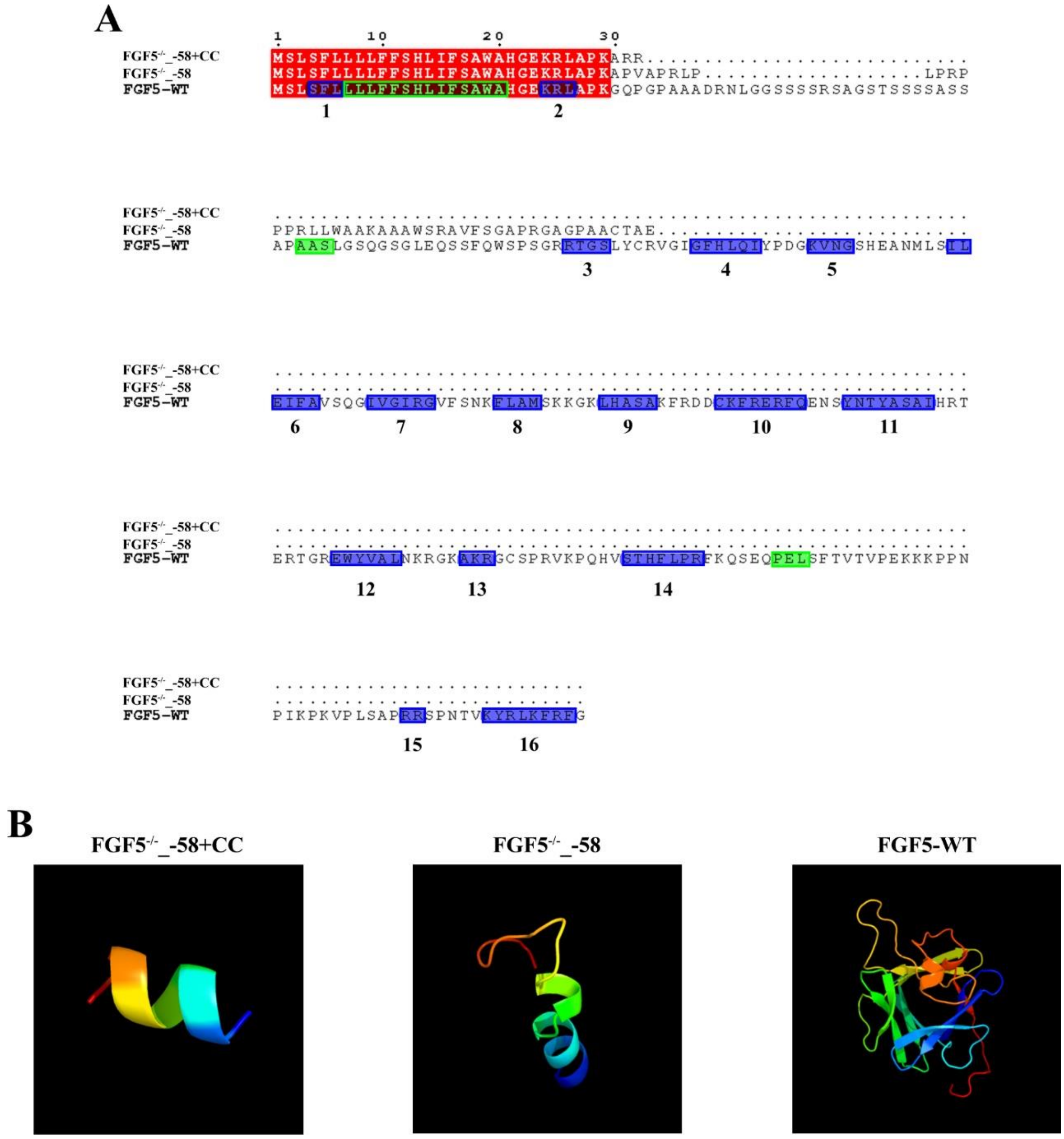

Figure S4 Prediction of the structural alteration among predominant FGF5-targeted and WT proteins.

A) Alignment of amino acid sequences of truncated FGF5, WT FGF5 and WT FGF5s proteins. Residues that are identical are shown in red. The location of 16 β-stands and 3 alpha-helices in the FGF5 WT protein, which are predicated by PHY2 software, are highlighted in blue and green, respectively. B)-E) 3D models built by the Phyre serve represent the WT FGF5, WT FGF5s and loss-of-function FGF5.

Tables

Table S1. Generation of genetically targeted rabbits using CRISPR/Cas9

	gRNA/Cas9 mRNA(ng/uL)	Embryos injected	Transferred embryos (% microinjected)	Recipients	Pups obtained (%transferred)	mutant transmission (%pups)
1	40/200	42	32(76.2%)	YES	8(25%)	2(25%)
2	40/200	37	35(94.6%)	NO		
3	40/200	38	33(86.8%)	NO		
4	40/200	40	37(92.5%)	NO		

Table S2: Primers used in PCR and QPCR.

Name	Primers	Sequence (5'-3')	Produce size(bp)
sgRNA1	sgRNA1-F	TAGGACGAGGTGCTACCGGCGCTC	24
	sgRNA1-R	AAACGAGCGCCGGTAGCACCTCGT	
sgRNA2	sgRNA2-F	TAGGAGAAGCGCCTCGCCCCCAAA	24
	sgRNA2-R	AAACTTTGGGGGCGAGGCGCTTCT	
FGF5	FGF5-F	AGAATGAGCTTGTCCTTCC	333
	FGF5-R	GCCATTGACTTTGCCATC	
FGF5-Q-PCR	F5-Q-F	ATCTACCCGGATGGCAAAG	101
	F5-Q-R	CTCCTCGTATTCCTACAATCCC	
TGF-β-Q-PCR	T-Q-F	GACACCCAGTACAGCAAGG	111
	T-Q-R	GTAGTACACGATGGGCAGTG	
GAPDH-Q-PCR	G-Q-F	TTCCACGGCAGGTCAAGGC	99
	G-Q-R	GGGCACCAGCATCACCCCAC	
VERSICAN-Q-PCR	V-Q-F	CTTATGTAGACACTGCCCATACC	113
	V-Q-R	TTGAGAGGGCTGACCTAGAA	
BMP2-Q-PCR	B2-Q-F	GATCACCTGAACTCCACTAACC	124
	B2-Q-R	CATCAAGGTACAGCATGGAGATA	
BMP4-Q-PCR	B4-Q-F	CCTGGTCAACTCTGTCAACTC	104
	B4-Q-R	GTACCACCTTGTCATACTCATCC	

Table S3. 8 potential off-target sites examined by PCR and primers used for list.

	Potential Off Target Site	Number of mismatches	Position	PCR Primer
S1	GGGGGTCCTCATTGCCCTTT	4	chrUN0: +2082728	F: CCTCTGCCCTGTCCAATTT<br>R: GGATACAGTTAGAAGCCGTAAGAG
	TGGGGTCCAGGTTGCCTTTT	4	chrUN0: +208274	F: CCAGAGTGCTGTCACTCATATAAT<br>R: GAATCCCAGAAGAGATCGGAAG
	GCGGGTCCGCCTTGCCCTCT	3	chrUN0: +477181	F: CGTAGTAGGTAGCCTTGATTGG<br>R: CAGCAGTTTGCATGTGTGAG
	GTGGGGCAGGGTTGCCCTTG	4	chr19: -42656111	F: CTCCTCGATGGGCTTGAAAT<br>R: CCAGGAAGCTGTCTGCTTTA

S2	ACCAGGTTCTACCGGCCCTC	3	chr12: +148165087	F: GTGAGGAAGATGGACTGAGATTT<br>R: ACATTAGCCCAGCTGGTATTT
	GGGAGGTGCGCCCGGCGCTC	4	chr21: +8975184	F: TGAGGTTCTGAGTTCCGGTAT<br>R: CCAAGTGTTCGCTTTCATGTTG
	ACCACGTGCACCCGGCGCTC	4	chr21: +8975184	F: CCCATCTGCTGGCTTACTTTA<br>R: GAGGCAGACAGGATAGAAACAG
	AAGAGGGGCGACTGGCGCTC	4	chr14: -448996	F: GCAGTTATCCCTCCCTGAATAG<br>R: CCTAGCTTTGCAACACCATTT
